# Sustainable Rubberized Concrete-Filled Square Steel Tubular Columns Under Eccentric Compression

**DOI:** 10.3390/ma19020250

**Published:** 2026-01-08

**Authors:** Yanhua Liu, Yong Bao, Senyan Jiang, Qingxin Ren, Yu Liu, Tong Li

**Affiliations:** 1 School of Civil Engineering and Transportation, Foshan University, Foshan 528225, China; liuyanhua@fosu.edu.cn (Y.L.); renqingxin@fosu.edu.cn (Q.R.); 2 College of Water Conservancy, Shenyang Agricultural University, Shenyang 110866, China; 17865816058@163.com (Y.B.); jsy573@163.com (S.J.); 3 School of Civil Engineering, University of Science and Technology Liaoning, Anshan 114051, China; liuyu1225@ustl.edu.cn

**Keywords:** rubberized concrete, concrete-filled steel tube, eccentric compression, rubber replacing ratio, slenderness ratio

## Abstract

This study examined rubberized concrete-filled steel tubular (RuCFST) columns as a sustainable option for structural applications. Eccentric compression tests were conducted on eight groups of square specimens, with two identical specimens per group. The main parameters were slenderness ratio, load eccentricity, and rubber replacement level for fine aggregates. Full load–displacement and load-strain curves were obtained. Results indicated that rubber particles inhibit concrete cracking. Increasing slenderness ratio reduces bearing capacity, with ductility peaking at moderate slenderness. Eccentricity significantly degrades bearing capacity and stiffness. A higher rubber replacement ratio lowers capacity but optimizes particle interaction and distribution, leading to stiffness recovery at higher ratios. Filling the steel tube with core concrete transforms it into a composite member, substantially improving load-bearing performance. Comparisons with seven design standards (including GB 50936-2014, CECS 254:2012, Eurocode 4, and AISC 360-16) revealed that Eurocode 4 provided the most reliable predictions, whereas AISC was the most cautious. None of the codes accounts for the effect of rubber on core concrete behavior. These results offer useful guidance for incorporating recycled rubber particles into composite columns to promote sustainable building practices.

## 1. Introduction

Waste rubber has become a prominent challenge in global solid waste management due to its resistance to degradation. Not only does stockpiling or landfilling use up a lot of land, but it also poses fire risks and the possibility of soil and water contamination [1]. Processing waste tires into crumb rubber to partially replace natural aggregates in concrete represents an effective strategy for high-value waste utilization, reducing the extraction of natural sand and gravel, and promoting the development of greener construction materials [2,3]. The utilization of industrial by-products such as steel slag, fly ash, and granulated blast furnace slag in concrete production has provided a successful model for the resource recovery of such solid wastes [4,5,6,7,8]. Following this approach, the large-scale application of crumb rubber as another typical solid waste-derived aggregate in load-bearing structures holds comparable significance for environmental protection and resource conservation. 

Research indicates that concrete incorporation of crumb rubber typically offers additional toughness and enhanced ductility with greater deformation capacity (particularly in the plastic range) relative to conventional concrete. However, its strength and elastic modulus are significantly reduced, with the extent of reduction strongly dependent on the rubber replacement ratio and the substituted aggregate type (fine or coarse). The lower elastic modulus contributes to the improved overall deformation performance by allowing greater strains under load. These favorable deformation characteristics have also been demonstrated in structural applications of rubberized concrete beyond confined composites, such as in pavements, tunnel engineering, and beams, where improved ductility, energy dissipation, and impact resistance are observed despite reductions in strength [9,10,11]. To overcome these strength limitations while fully exploiting the favorable deformation characteristics of rubberized concrete, confining it within steel tubes to form rubberized concrete-filled steel tubular (RuCFST) members has been proposed. The ductility, earthquake resistance, and crack resistance of the composite member can all be significantly increased by the strong lateral confinement provided by the outer steel tube [12,13,14,15,16,17].

Extensive research has been conducted on the mechanical behavior of conventional concrete-filled steel tubular (CFST) members under axial compression [12], eccentric compression [13,14], pure bending [15], and cyclic loading [16], as well as on their interfacial bond-slip behavior [17]. These studies form the basis of current design codes. In recent years, RuCFST members have attracted increasing attention. Experimental studies on axial compression, bending, and cyclic loading conducted by Duarte et al. [18], Silva et al. [19], Elchalakani et al. [20], Yang et al. [21], and others have demonstrated that RuCFST members can achieve load-bearing capacities comparable to those of conventional CFST members. However, their ductility and energy dissipation performance exhibit variable trends contingent upon rubber content and replacement strategy: replacement ratios of 10% or lower, or the substitution of coarse aggregates, frequently yield enhanced or comparable ductility, whereas fine aggregate replacement ratios of 15% or higher generally lead to diminished ductility due to the substantial reduction in elastic modulus and the compromised interfacial transition zone between rubber aggregates and the cementitious matrix [22].

Despite these advances, most existing studies have focused primarily on concentric or low-eccentricity loading conditions. However, in real structures, columns are almost inevitably subjected to eccentric compression due to construction imperfections, unintentional load eccentricity, or additional moments induced by wind or seismic actions. So far, systematic experimental investigations remain scarce on the failure modes, load-carrying capacity, and especially ductility of RuCFST members under eccentric compression, particularly when crumb rubber replacement ratios (*r* = 10% and 20% by volume of fine aggregate) are combined with varying slenderness ratios (*λ* = 40, 60, and 80) and load eccentricities (*e*_0_ = 0, 20, and 40 mm). This critical knowledge gap currently hinders the confident large-scale application of waste rubber in load-bearing composite structures.

To address this research gap, the present study conducted eccentric compression tests on square RuCFST columns with *r* of 10% and 20%. Key parameters, including slenderness ratio (*λ*), load eccentricity (*e*_0_), and the presence or absence of core concrete, were systematically investigated. The applicability of major existing design codes for predicting the ultimate bearing capacities (*N*_ue_) of RuCFST members was also evaluated. This research provides crucial experimental evidence for the large-scale application of waste rubber in load-bearing composite structures and offers valuable insights for the development of green seismic design methodologies and dedicated design specifications.

## 2. Experimental Details

### 2.1. Test Specimens

**Figure 1:**
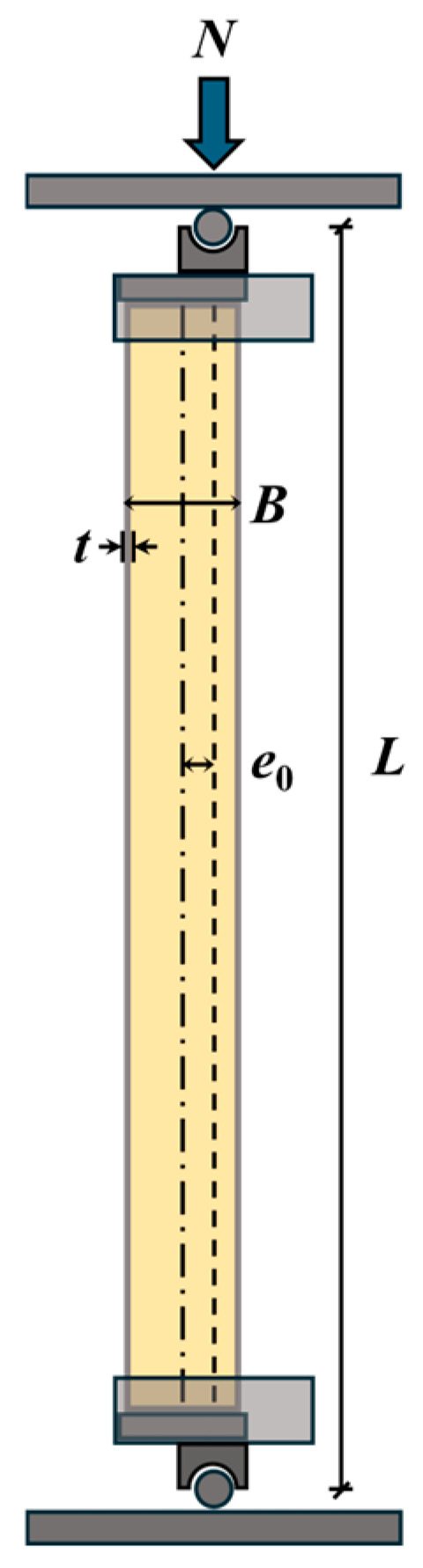
Schematic diagram of the test specimen showing the cross-section, dimensions, and loading eccentricity *e*_0_.

A total of eight groups of square specimens were designed and fabricated, with two identical replicates in each group to ensure test repeatability. This included six groups of RuCFST columns (src1–src6) with rubber replacement ratios of 10% and 20% (by volume of fine aggregate), one group of conventional CFST columns (src7, *r* = 0%), and one group of hollow steel tubes (src8, without core concrete). The schematic diagram of the test specimens, including the eccentricity *e*_0_, is shown in Figure 1. The detailed specimen parameters and labeling are presented in Table 1, where *B* and *t* denote the outer side length and wall thickness of the square steel tube, respectively, and *L* represents the effective height of the specimens.

The *λ* was calculated based on the geometric proportion *λ* = 4*L*/*B* for parametric design, where *L* is the effective height and *B* is the outer side length of the steel tube. This nominal slenderness ratio is commonly used in studies on square CFST columns to facilitate comparison with circular sections and does not involve the radius of gyration of the composite section or transformation of the concrete core into equivalent steel for moment of inertia calculation.

The investigated parameters were *λ* (40, 60, and 80), *e*_0_ (0, 20, and 40 mm), *r* (0, 10%, and 20%), and the presence of core concrete. The λ was assigned only to the concrete-filled specimens (src1–src7), while the hollow steel tube specimen src8 was not assigned a *λ* value, as its stability is primarily governed by local buckling rather than overall buckling. The selected range of *λ* (40, 60, and 80) covers typical intermediate to slender square CFST columns under eccentric compression, allowing clear observation of the progressive influence of overall buckling on load-bearing capacity, ductility, and failure modes as increases. The test matrix was systematically designed such that groups src1–src3 primarily varied the *λ*, groups src1, src4, and src5 focused on the effect of *e*_0_, groups src1, src6, and src7 highlighted the influence of *r*, whereas groups src1, scr7, and src8 were compared to assess the contribution of concrete infill. To focus on typical eccentric loading (*e*_0_ = 40 mm), six groups were tested under this condition, while only one group each was used for *e*_0_ = 20 mm and *e*_0_ = 0 mm as supplementary comparisons.

All specimens except src5 (*e*_0_ = 0 mm, axial compression) were subjected to eccentric compression. The tested eccentricity ratios *e*_0_/*B* ranged from 0 to 0.286, covering axial compression, small eccentricity (*e*_0_/*B* = 0.143), and moderate eccentricity (*e*_0_/*B* = 0.286), which are representative of typical loading conditions in engineering practice.

### 2.2. Material Properties

The square steel tubes were fabricated by welding four steel plates. A 10 mm-thick square end plate was welded to each end of the tubes. The properties of the steel obtained from tensile tests are provided in Table 2.

**Figure 2:**
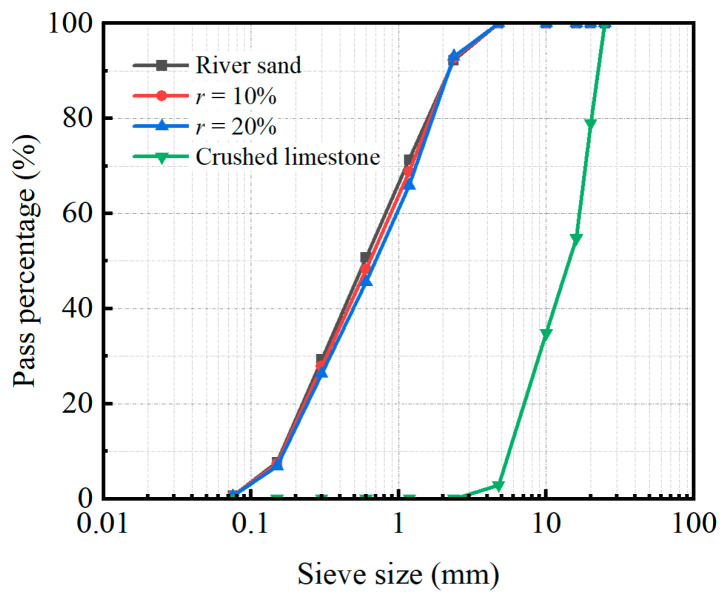
Particle size distribution of aggregates and rubberized mixtures (0%, 10%, 20% replacement).

The concrete materials used in this test consisted of conventional concrete and rubberized concrete, with the latter incorporating rubber at replacement ratios of 10% and 20% by volume of fine aggregate. Ordinary Portland cement with 28-day compressive strength of 42.5 MPa and Grade II fly ash were employed as cementitious materials. The coarse aggregate consisted of crushed limestone with a maximum particle size of 20 mm, while natural river sand served as the fine aggregate. Crumb rubber particles (particle size: 1–2 mm) were used to partially replace the river sand by volume. The particle size distribution (PSD) curves of the natural river sand, fine aggregate mixtures with 10% and 20% rubber replacement, and the crushed limestone are shown in Figure 2.

The mixed proportions of the concrete are detailed in Table 3. Although the rheological properties of the fresh rubberized concrete were not experimentally investigated in this study, as the primary focus was on the hardened mechanical behavior of RuCFST columns, previous studies have indicated that 10–20% volume replacement of fine aggregate with 1–2 mm crumb rubber particles moderately reduces workability due to increased friction, but this can be effectively compensated by increasing the superplasticizer dosage to ensure satisfactory flowability for casting [23].

To determine the mechanical properties, cube and prism specimens were prepared and cured under the same conditions as the RuCFST columns. After casting, all specimens and RuCFST columns were covered with plastic sheets and left in the laboratory at ambient temperature (approximately 20 ± 3 °C) for 24 h. They were then demolded and cured in a standard curing room with a temperature of 20 ± 2 °C and relative humidity above 95% until the age of 28 days. Compression tests were conducted in accordance with the Chinese standard GB/T 50081 [24]. The measured 28-day cubic compressive strength (*f*_cu_), prismatic compressive strength (*f*_c_^′^), and elastic modulus (*E*_c_) are summarized in Table 3.

### 2.3. Test Setup and Instrumentation

**Figure 3:**
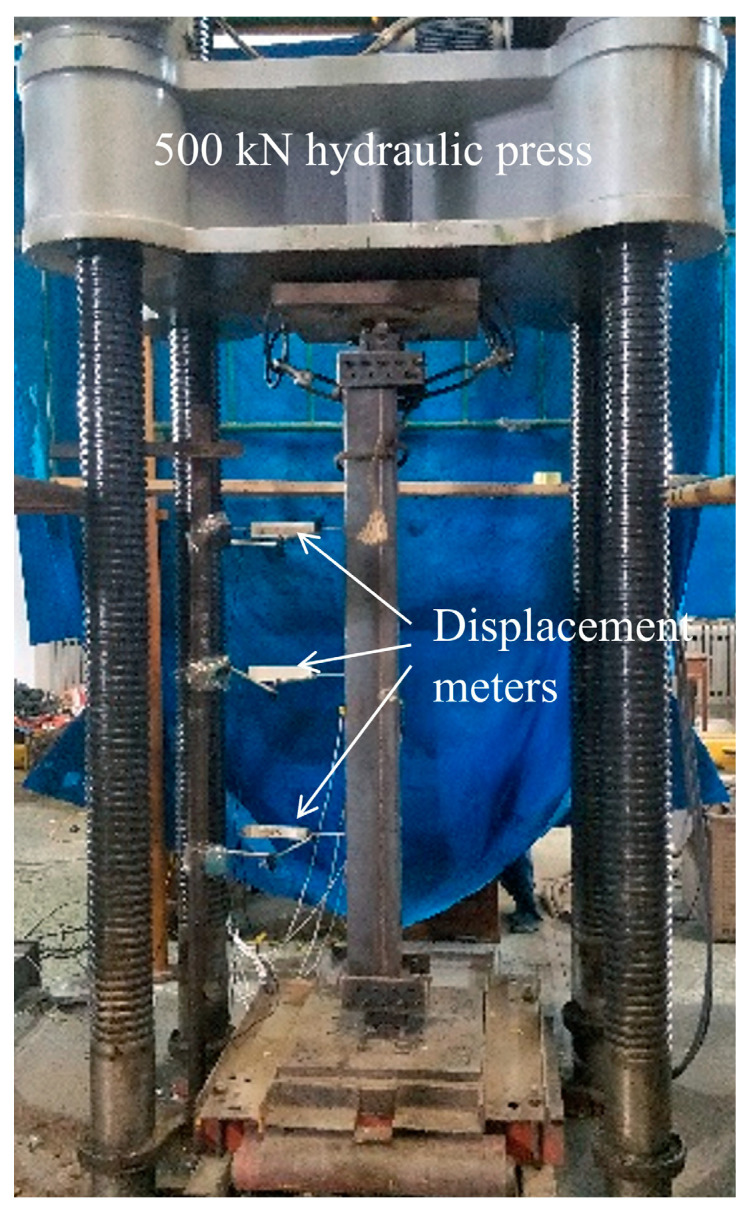
Setup for compression test and lateral deformation measurement.

The tests were conducted using a 500 kN electro-hydraulic servo compression machine (Changchun Testing Machine Research Institute, Changchun, China). compression machine. Prior to loading, both ends of the specimens were ground flat to ensure uniform bearing. The specimens were tested with pinned-ended conditions in the eccentricity plane. Cylindrical roller hinges were used at both ends to permit rotation in the bending plane while preventing out-of-plane rotation and lateral movement (see Figure 1). This arrangement gives an effective length factor *K* ≈ 1.0, which matches the nominal slenderness ratio *λ* = 4*L*/*B* adopted in this study. The applied load was measured with a load cell into the compression machine (i.e., the aforementioned 500 kN electro-hydraulic servo system). Strains were monitored using electrical resistance strain gauges (Aideli Electric Measurement Co., Ltd., Dongguan, China). A total of five pairs of strain gauges were attached to the surfaces of the square column: one pair on the tensile face, one pair on the compressive face, and the remaining three pairs evenly distributed on the other two opposite sides. For deformation measurement, three displacement meters were mounted on the supporting columns to capture lateral deflection, as illustrated in Figure 3. Additionally, three displacement transducers were installed along the specimen height at the following vertical positions: the upper-third point, mid-height, and the lower-third point, to measure axial shortening. All data from the strain gauges and displacement transducers were automatically recorded by an isolated measurement pods (IPM) data acquisition system. All reported results are the average values of the two identical specimens in each group, with variations generally within 10%, confirming good repeatability.

### 2.4. Loading Procedure

A hierarchical loading protocol was adopted. Prior to formal testing, a preload of 15% of the predicted peak load was applied, held for 2 min, and then unloaded. During the formal phase, loading was displacement-controlled at a constant rate of 0.5 mm/min. Within the elastic range, the load increment per step did not exceed 1/10 of the predicted peak load. After yielding, the increment was reduced to less than 1/20 of the predicted peak load. Each loading step was maintained for 2 min.

The loading was continued into the post-peak descending branch, and tests were terminated when the load fell to 80% of the peak load, excessive deformation occurred (axial displacement 50–60 mm or mid-height deflection > *L*/30), or severe local buckling prevented further loading.

### 2.5. Parametric Equations and Analysis Basis

To quantitatively evaluate the effects of the test parameters, specimen src1 (*λ* = 40, *e*_0_ = 40 mm, *r* = 20%,with core concrete) was selected as the benchmark specimen. Following the widely adopted parametric analysis framework proposed by Han et al. [25], three dimensionless performance indices normalized to src1 were defined as:

(1) $$SI=\frac{N_{\mathrm{ue}}}{N_{\mathrm{ue},\mathrm{scr}1}}$$

(2) $$RI=\frac{K_{\mathrm{ini}}}{K_{\mathrm{ini},\mathrm{scr}1}}$$

(3) $$DI=\frac{\mu }{u_{\mathrm{src}1}}$$

where *N*_ue_ and *N*_ue,src1_ are the ultimate bearing capacities of the current specimen and the benchmark specimen src1, respectively; *K*_ini_ and *K*_ini,src1_ are the initial stiffnesses of the current specimen and src1, respectively, determined as the secant stiffness corresponding to 40% of *N*_ue_ on the ascending branch of the load–displacement curve; *μ* = *δ*_0.85_/*δ*_μ_ and *μ*_src1_ = *δ*_0.85,src1_/*δ*_μ,src1_ are the displacement ductility factors, in which *δ*_μ_ is the mid-height lateral displacement at the *N*_ue_, and *δ*_0.85_ is the mid-height lateral displacement when the load drops to 85% of *N*_ue_ in the post-peak branch.

By normalizing *SI*, *RI*, and *DI* to the same reference specimen src1, the effects of *λ*, *e*_0_, *r* and the presence or absence of core concrete can be directly and consistently compared. This relative quantification approach is extensively used and validated in parametric studies of CFST members.

## 3. Test Results and Evaluations

### 3.1. Failure Mode

**Figure 4:**
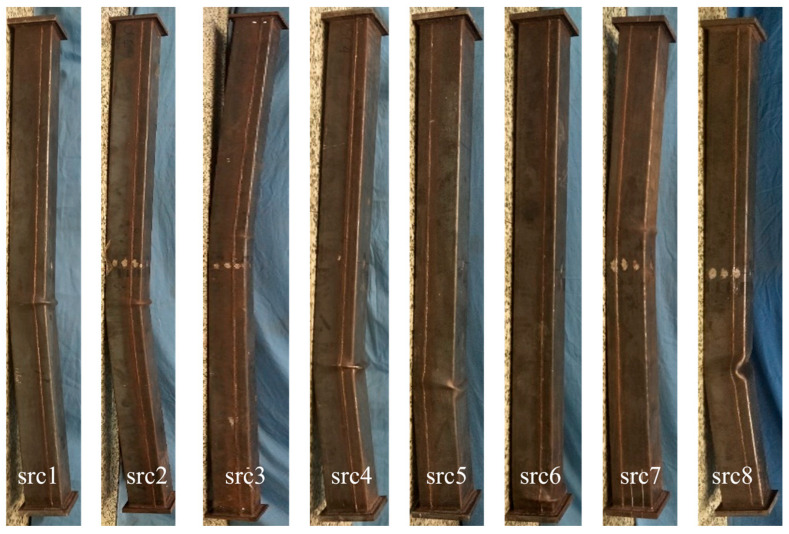
Typical failure patterns of the specimens under axial compression.

Figure 4 presents the ultimate failure modes of all specimens under eccentric compression. Experimental observations revealed that the failure mechanisms of RuCFST specimens and conventional CFST specimens were remarkably similar, with local buckling and outward bulging consistently observed in all square steel tubes and no apparent regularity in bulge distribution; notably, the hollow tube specimen (src8) exhibited significantly greater deformation due to the absence of core concrete confinement. Macroscopic examination confirmed high consistency in failure modes across the series, with variations in *λ*, *e*_0_, and *r* exerting negligible influence on overall morphology, thus underscoring the dominance of local steel tube buckling in governing failure under eccentric loading. This behavior stems from the synergistic interaction between the steel tube and core infill under combined axial and flexural stresses. Although rubber particles reduce the elastic modulus and compressive strength of the core concrete, the effective lateral confinement provided by the steel tube compensates for this degradation, thereby preserving global stability and failure characteristics comparable to those of CFST members.

However, slight differences were observed in a few specimens: src5 (*e*_0_ = 0 mm) exhibited less obvious buckling, primarily because the bending stress was minimal under axial compression, leading to a failure mode more inclined toward overall crushing of the core concrete; src6 (*r* = 10%) and src7 (*r* = 0) showed relatively milder local buckling, mainly due to the higher stiffness of the core concrete, which provided stronger support and delayed the development of steel tube deformation. These differences further confirm the influence of eccentricity and rubber content on the prominence of local buckling, but did not alter the overall failure characteristic of the series, which remained dominated by local buckling of the steel tube.

**Figure 5:**
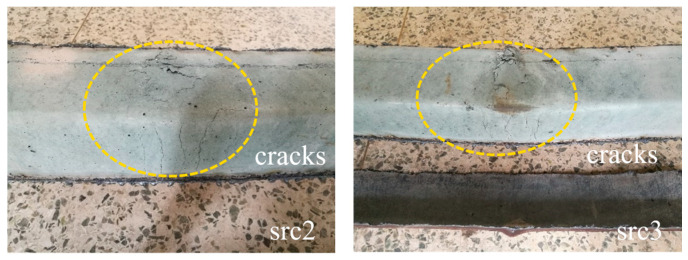
Typical failure modes of the core concrete exposed after removal of the steel.

Figure 5 displays the failure morphology of the core concrete in specimens src2 and src3 after being longitudinally sectioned post-testing. Although concrete crushing and surface spalling occurred in the compression zone, corresponding to the outward bulging of the steel tube, and sparse transverse cracks with a propagation length of approximately half the section width developed in the tension zone, the core concrete maintained satisfactory structural integrity. The observed cracks were characterized by fine widths and a uniform distribution. These relatively moderate damage characteristics, particularly the restrained crack propagation in the tension zone, suggest that the incorporated rubber particles may have effectively inhibited the initiation and development of cracks within the core concrete through bridging effects and an energy dissipation mechanism. The underlying mechanism can be attributed to the elastic deformation capability of the rubber aggregates, which enables them to accommodate tensile strain and redistribute stress concentration at the crack tips, thereby enhancing the fracture toughness of the concrete.

### 3.2. Load–Displacement Curves

**Figure 6:**
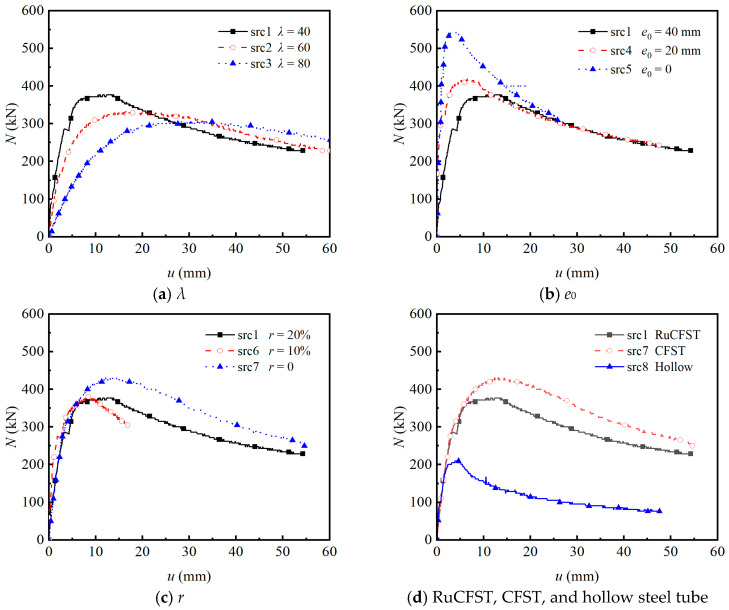
Load–displacement curves of the test specimens.

The axial load versus mid-height lateral displacement (*N*-*u*) curves of all specimens under varying test parameters are illustrated in Figure 6, with each curve clearly exhibiting three distinct stages. As the *λ* increases (Figure 6a), both *N*_ue_ and stiffness decrease markedly, while midspan displacement rises rapidly. The displacement initially increases linearly with load, then accelerates beyond 70% to 80% of the ultimate load due to second-order effects, a phase where the growth rate of the second-order moment surpasses that of the section resistance moment. This is followed by a sharp post-peak surge in displacement as the load declines. A similar trend is observed with increasing *e*_0_ (Figure 6b), where the *N*_ue_ drops substantially and displacement grows continuously. As shown in Figure 6c, incorporating crumb rubber particles leads to a modest reduction in *N*_ue_, with the specimens at 10% and 20% replacement ratios exhibiting almost identical values. However, the specimen with 10% rubber replacement shows a significantly smaller ultimate mid-height lateral displacement and a steeper post-peak descending branch compared to the other mixtures, indicating markedly poorer ductility rather than enhanced ductility. Comparison of filled and hollow specimens in Figure 6d reveals negligible differences in the initial elastic stiffness. However, both rubberized (RuCFST) and normal concrete-filled (CFST) members exhibit markedly higher peak loads and superior post-peak behavior compared to hollow tubes, which fail prematurely due to early buckling. These findings are qualitatively consistent with previous studies on conventional CFST columns under eccentric compression [13,14].

### 3.3. Load-Strain Curves

**Figure 7:**
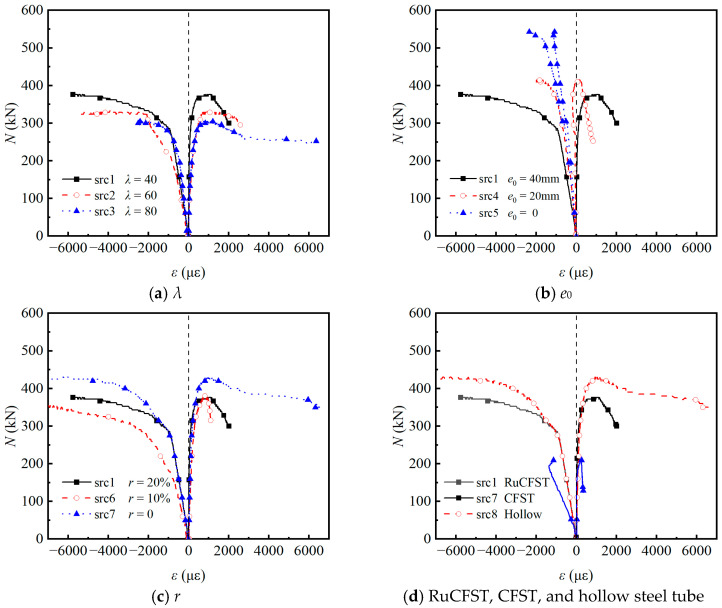
Load–strain curves of the test specimens.

Figure 7 presents the axial load versus longitudinal strain (*N*–*ε*) curves of the specimens on both the compressive and tensile sides, where positive values indicate tensile strain and negative values represent compressive strain. During the initial elastic stage, strains on both sides increased linearly with the applied load, consistent with predictions from classical beam theory regarding uniform stress distribution in short-to-medium columns under combined axial and bending actions. This linear phase reflects the composite elastic modulus of the rubberized concrete core and steel tube before significant yielding or local buckling occurs. As shown in Figure 7a, the *N*_ue_ decreased with increasing *λ*, primarily due to the greater susceptibility of slender members to second-order effects and global buckling. Concurrently, the increase in peak tensile strain indicates enhanced deformation capacity prior to failure, a characteristic behavior of slender compression members where geometric imperfections play a more dominant role. Figure 7b further illustrates that the *N*_ue_ decreased progressively with increasing *e*_0_. Under axial loading (*e*_0_ = 0 mm), the specimen remained entirely in compression throughout the test. In contrast, for *e*_0_ = 20 mm, the tensile side initially exhibited compressive strain but transitioned to tension as loading continued, indicating a shift in the neutral axis driven by bending moments. This transition signifies the onset of tension–compression coupling and a reduction in the effective compression zone, accelerating yielding on the tensile side. The incorporation of rubber powder reduced both *N*_ue_ and ductility, as seen in Figure 7c. With increasing rubber replacement ratio, the post-peak descent in the *N*–*ε* curves became steeper, indicating decreased deformability and more rapid loss of load-carrying capacity after the peak. This steeper post-peak behavior provides new evidence, compared to most existing studies on rubberized concrete-filled steel tubes that typically report improved ductility under concentric compression because of the distinct response under combined axial and bending loading [26]. The rubber particles, although more elastic than the cementitious matrix, did not sufficiently hinder microcrack propagation under the present confinement and loading conditions to prevent earlier brittle-like failure of the core. This finding aligns with the rubberized concrete but highlights a reduction in ductility in this specific structural configuration, similar to observations in bond performance tests where ductility reduced with higher rubber incorporation [18].

Finally, Figure 7d demonstrates that hollow steel tubes exhibited significantly lower *N*_ue_ and failure strain compared to filled specimens. This highlights the fundamental composite mechanism in filled tubes: the concrete core (both normal and rubberized) restrains inward local buckling of the steel tube, while the steel tube provides confinement to the concrete, enhancing its strength and ductility through triaxial stress states. The absence of this synergistic interaction in hollow sections leads to premature buckling and inferior structural performance.

### 3.4. Parametric Sensitivity Analysis

**Figure 8:**
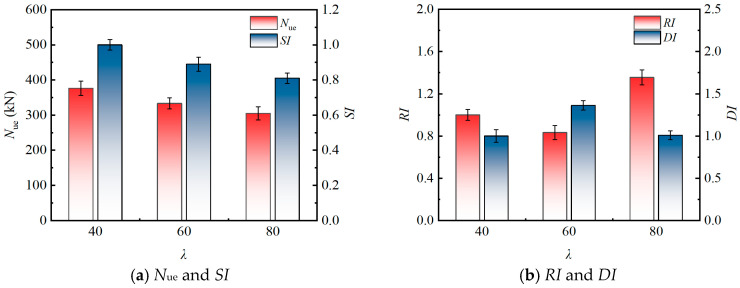
Influence of *λ* on the eccentric compression behavior.

**Figure 9:**
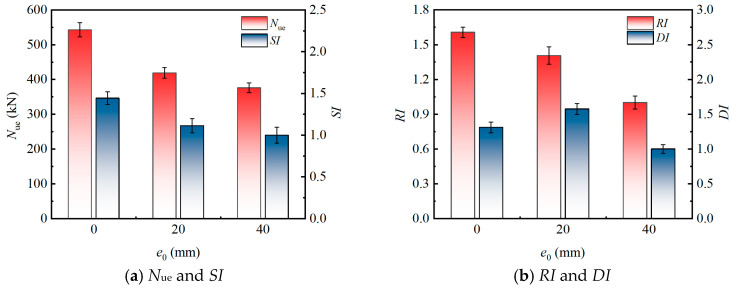
Influence of *e*_0_ on the eccentric compression behavior.

**Figure 10:**
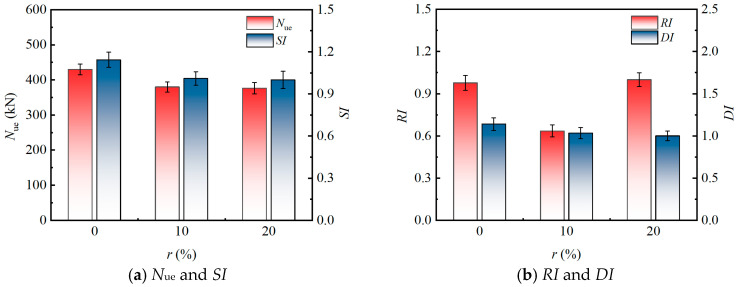
Influence of *r* on The Eccentric Compression Behavior.

**Figure 11:**
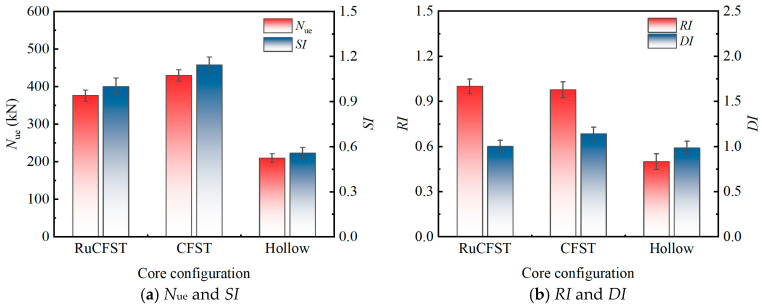
Influence of concrete infill type on the eccentric compression behavior.

The following parametric analysis (Figure 8, Figure 9, Figure 10 and Figure 11) is primarily based on the six specimen groups tested under moderate eccentricity (*e*_0_ = 40 mm): src1–src3 (varying *λ*), src1, src6, src7 (varying *r*), and src8 (hollow tube). The effects of eccentricity are supplemented by src4 (*e*_0_ = 20 mm) and src5 (*e*_0_ = 0 mm).

#### 3.4.1. Effect of Slenderness Ratio (λ)

The influence of the *λ* on the eccentric compression performance of the specimens is shown in Figure 8 (based on three specimen groups: src1, src2, and src3). As *λ* increased from 40 to 80, the *N*_ue_ exhibited a continuous declining trend, dropping from 376.19 kN to 304.76 kN, with a cumulative decrease of approximately 19.0%. This phenomenon aligns with classical steel structure stability theory, primarily stemming from the enhanced second-order effects induced by the increased slenderness ratio, which reduces member stability and leads to a reduction in bearing capacity. In terms of performance indicators, the *SI* decreased monotonically with increasing *λ*, further confirming the bearing capacity trend. In contrast, the *RI* and *DI* exhibited non-monotonic trends, which is characteristic of RuCFST columns due to the significantly enhanced ductility and deformation capacity provided by the rubberized concrete core. This allows better resistance to slenderness-induced degradation compared to conventional CFST columns [27].

#### 3.4.2. Effect of Eccentricity Distance (e_0_)

The influence of *e*_0_ on eccentric compression performance of the specimens is presented in Figure 9 (based on three specimen groups: src1, src4, and src5). A significant reduction in the *N*_ue_ was observed with increasing *e*_0_ (from 0 to 40 mm, corresponding to normalized eccentricity ratios *e*_0_/*B* = 0 to 0.286). Specifically, the *N*_ue_ of specimen src1 (*e*_0_ = 40 mm) was 10.2% and 30.7% lower than that of specimens src4 (*e*_0_ = 20 mm) and src5 (*e*_0_ = 0 mm), respectively. This degradation in bearing capacity is primarily due to the additional bending moment induced by eccentric loading. The increased eccentricity leads to a non-uniform stress distribution across the cross-section, causing early cracking of the rubberized concrete on the tensile side and potential local buckling of the steel tube on the compressive side, thereby accelerating failure.

Both the *SI* and *RI* decreased as the *e*_0_ increased. Notably, when *e*_0_ increased from 0 mm to 40 mm, the *RI* exhibited a more substantial decrease (37.8%) compared to the *SI* (30.7%), highlighting a greater sensitivity of stiffness to *e*_0_. This can be attributed to their distinct mechanical origins: the *SI* is more influenced by the peak strength of composite material, governed by steel tube confinement and rubber particle energy absorption, while the *RI* is directly tied to the initial elastic modulus and moment of inertia, which are more severely compromised by bending effects. Furthermore, the *DI* demonstrated a non-linear response. It increased from 1.309 to 1.575 as the *e*_0_ rose from 0 mm to 20 mm (small eccentricity), suggesting that a moderate eccentricity can enhance deformation capacity and energy dissipation, likely due to the beneficial role of rubber particles in mitigating crack propagation. However, a further increase in eccentricity to 40 mm (moderate eccentricity) caused the *DI* to drop to 1.000. The tested range primarily covers axial compression to moderate eccentricity (*e*_0_/*B* ≤ 0.286), which is typical in practical engineering scenarios. This decline indicates that large eccentricities promote pronounced stress concentration and asymmetric damage patterns, which ultimately overwhelm the confinement system and lead to a more brittle failure mode.

#### 3.4.3. Effect of Rubber Replacing Ratio (r)

Figure 10 illustrates the influence of the *r* on eccentric compression performance of the specimens (based on three specimen groups: src1, src6, and src7). The incorporation of rubber particles generally reduces the *N*_ue_ of the specimens. At *r* = 10%, the *N*_ue_ decreased by approximately 12.5% compared to the specimen without rubber. However, when the *r* increased to 20%, the *N*_ue_ showed no further significant reduction and remained nearly identical to that of the specimen with *r* = 10%. This nonlinear variation stems from the flexible filling effect of rubber particles. At low replacement ratios, the substitution of aggregate with rubber leads to reduced compactness and strength loss. In contrast, at the higher replacement ratio of 20%, a more uniform distribution optimizes stress transfer, partially counteracting the strength attenuation. Under eccentric loading, although rubber particles absorb energy, their low elastic modulus amplifies non-uniform deformation across the section, resulting in an initial reduction in *N*_ue_. The subsequent stabilization reflects the adaptive characteristics of the composite material. Regarding strength performance, the *SI* exhibited a decreasing trend with increasing rubber content. Specifically, the *SI* of specimen src1 (*r* = 20%) was 12.5% lower than that of the benchmark specimen src7 (*r* = 0%), but only 1.0% lower compared to src6 (*r* = 10%). This trend indicates that rubber replacement primarily diminishes the peak strength at low to medium replacement ratios. This is attributed to the low-strength characteristics of rubber particles, which interfere with the confining effect of the steel tube on the core concrete and reduce the effective load-bearing area of the composite section.

Concerning the *RI*, as the *r* increased, the *RI* demonstrated an initial decline followed by a subsequent rise. When the *r* increased from 0% to 10%, the *RI* decreased from 0.977 to 0.635, indicating that the introduction of rubber significantly reduced the initial elastic stiffness. This reduction is primarily due to the decreased sectional moment of inertia and the propagation of early-stage microcracks resulting from the low modulus of elasticity of rubber. However, when the replacement ratio increased to 20%, the *RI* recovered to the benchmark value of 1.000. This recovery may be due to better overall stability caused by particles locking together at high rubber content, which partially restores stiffness. When rubber is under an eccentric load and has a low replacement ratio, it is more likely to deform in a localized way, which speeds up the loss of stiffness. A higher replacement ratio, on the other hand, solves this problem by improving the way energy is dissipated.

Furthermore, as the *r* increased from 0% to 10% and then to 20%, the *DI* decreased by 9.4% and 12.3% compared with the specimen without rubber particles (*r* = 0%), respectively. Many studies have indicated that rubber particles can improve ductility in plain concrete or in members with weak confinement. In contrast, current experimental results indicate a moderate decline in *DI* as rubber content rises under eccentric compression. This unexpected trend mainly results from the strong confinement offered by the square steel tube, which fundamentally changes the behavior of rubber particles. In standard concrete-filled steel tubes without rubber, the steel tube restrains cracking in the core concrete and delays local buckling, producing a fairly ductile response after the peak load.

When rubber particles are added, they lower the elastic modulus and reduce interfacial friction in the core concrete. This weakens the interaction between the concrete core and the steel tube. The main reason for the reduced confinement lies in the much lower stiffness and Poisson’s ratio of rubber compared with natural aggregates. Ordinary concrete expands laterally under compression because of the Poisson effect. This expansion generates passive confining pressure from the steel tube and increases the triaxial strength of the core. However, rubber particles are highly compressible and typically have a Poisson’s ratio of 0.45 to 0.49 (much higher than the roughly 0.20 value for normal concrete), so they restrict lateral expansion of the core. The steel tube therefore develops lower confining stress, leading to weaker triaxial reinforcement and an overall drop in confinement effectiveness. Under eccentric loading, these effects become more severe. The lower lateral pressure intensifies uneven stress distribution across the section, causing earlier debonding at the interface and greater second-order effects. As a result, interface slippage starts sooner, the load–displacement curves show a sharper drop after the peak, and post-peak deformation capacity decreases at the standard *δ*_0.85_ level. All these factors contribute to the observed lower *DI* values.

The observed trend is consistent with several recent studies on rubberized concrete-filled steel tubular members under combined compression and bending [28], where moderate rubber contents (10–20%) were found to impair rather than enhance ductility when strong external confinement dominates the structural response. These findings highlight that, in squarely confined RuCFST columns subjected to eccentric compression, the beneficial energy-absorption characteristics of rubber particles are partially suppressed by the confinement-induced stress state, emphasizing the need to determine an optimal replacement ratio beyond the range investigated herein to achieve ductility enhancement.

#### 3.4.4. Effect of Core Concrete Infill

Figure 11 illustrates the influence of the core concrete infill type on eccentric compression performance of the specimens (based on three specimen groups: src1, src7, and src8). The results indicate that the presence of concrete infill (both conventional and rubberized) significantly improved both the load-bearing capacity and stiffness of the specimens compared to the hollow steel tube. Specifically, the conventional CFST specimen (src7) and the RuCFST specimen (src1) achieved ultimate loads *N*_ue_ of 430.00 kN and 376.19 kN, respectively, representing increases of 105.2% and 79.5% over that of the hollow steel tube specimens (209.52 kN). Correspondingly, the *SI* increased by 105.2% for src7 and 79.5% for src1, and the *RI* increased by 95.8% for src7 and 100.4% for src1, indicating that both types of concrete infill not only enhanced the load-carrying capacity but also effectively improved the deformation resistance of the members. In contrast, the *DI* increased by 16.1% for src7 but only 1.8% for src1 compared to the hollow tube, suggesting that the ductility enhancement from rubberized concrete was limited under eccentric compression.

The infill of concrete (with or without rubber) strengthened the composite action between the steel tube and the core concrete. The rubber particles in RuCFST enhanced the elastic deformation capability of the concrete, allowing it to better adapt to local deformations in the steel tube caused by Poisson effects and bending during the early loading stage. This reduced interfacial slip, which was key to the overall stiffness improvement. Simultaneously, the core concrete provided effective radial support to the steel tube wall, delaying the development of local buckling on the compression side and enabling the specimens to maintain higher residual load capacity after peak load. However, under eccentric loading, the shift in the neutral axis caused concrete in the tensile zone to crack and lose load-carrying function, resulting in a weaker confinement effect on the core concrete compared to that under axial compression. Under such conditions, the energy dissipation mechanisms related to friction and volumetric deformation of rubber particles were not fully activated. The post-peak deformation capacity mainly depended on the plastic hinge rotation formed after the local buckling of the steel tube on the compression side. This suggests that under eccentric compression, the contribution of rubberized concrete to ductility is primarily reflected in suppressing the early buckling of the steel tube and extending the post-peak plateau stage, rather than significantly increasing the ultimate deformation capacity. The bond-slip behavior between concrete and steel tubes is a key part of this composite action [29]. Future studies will look at how well rubberized concrete bonds and slips with steel tubes.

### 3.5. Applicability of Existing Design Provisions

#### 3.5.1. Chinese Design Provisions

**Figure 12:**
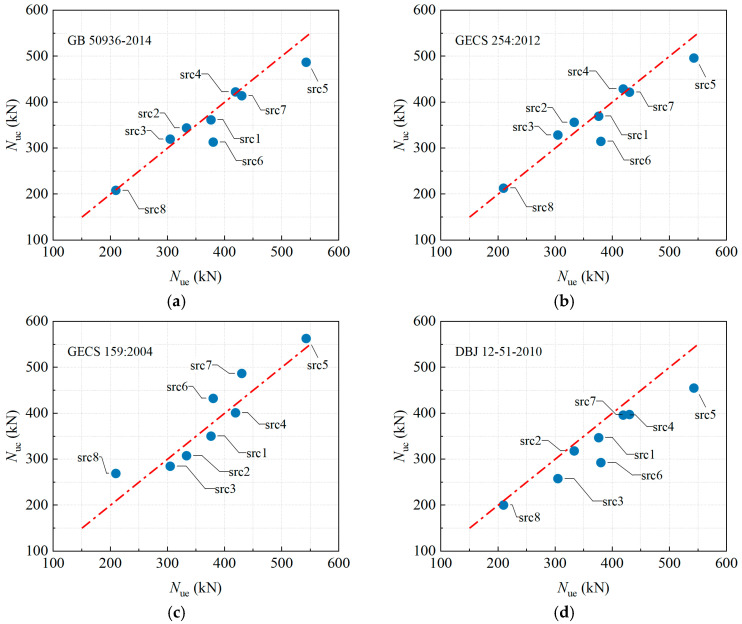
Comparison between calculated strength by different codes of China and test results. (**a**) GB 50936-2014 [30]. (**b**) CECS 254:2012 [31]. (**c**) CECS 159:2004 [32]. (**d**) DBJ 12-51-2010 [33].

To evaluate the applicability of current Chinese design codes for predicting the eccentric compression capacity of RuCFST columns, this study calculated the theoretical capacities using the formulas specified in GB 50936-2014 [30], CECS 254:2012 [31], CECS 159:2004 [32], and DBJ 13-51-2010 [33]. These are major Chinese specifications for CFST structures, all applicable to buildings, with differences mainly in confinement models and stability factors leading to varying conservatism. The calculated (*N*_uc_) and experimental ultimate bearing capacities (*N*_ue_), their ratios, and corresponding statistical measures are summarized in Table 4, with a visual comparison between the code predictions and test results provided in Figure 12.

The results indicate that GB 50936-2014 and CECS 254:2012 achieved the highest prediction accuracy, with mean *N*_uc_/*N*_ue_ ratios of 0.966 and 0.986 and corresponding coefficients of variation (COV) of 0.077 and 0.084, demonstrating minimal scatter and close agreement with tests. In contrast, CECS 159:2004 overestimated the capacity by 4.2% (mean *N*_uc_/*N*_ue_ = 1.042) with a higher COV of 0.127, indicating a tendency toward unsafe predictions. DBJ13-51-2010 provided the most conservative estimates, underestimating capacity by 10.6% (mean *N*_uc_/*N*_ue_ = 0.894). Further analysis of specimen parameters revealed the following trends:

As the slenderness ratio *λ* increased from 40 to 80, the *N*_uc_/*N*_ue_ ratios predicted by GB 50936-2014 and CECS 254:2012 gradually increased from 0.961 and 0.982 to 1.049 and 1.078, respectively. This indicates a shift from slightly conservative to slightly unsafe predictions. Meanwhile, the conservatism of DBJ13-51-2010 became more pronounced with increasing slenderness, with its *N*_uc_/*N*_ue_ ratio decreasing from 0.922 to 0.846.

When the initial eccentricity *e*_0_ increased from 0 mm to 40 mm, GB 50936-2014 and CECS 254:2012 maintained high robustness, with *N*_uc_/*N*_ue_ fluctuating within the ranges of 0.897–1.023. Conversely, CECS 159:2004 exhibited the most significant overestimation under zero eccentricity (*e*_0_ = 0 mm), where the ratio reached 1.037.

With an increase in the rubber aggregate replacement ratio *r* from 0% to 20%, the degree of overestimation by CECS 159:2004 decreased markedly, with its *N*_uc_/*N*_ue_ ratio dropping from 1.132 to 0.931. This suggests that the code’s reduction factor for the steel tube confinement effect is insufficient to account for the strength reduction in the core concrete caused by the rubber aggregates. DBJ13-51-2010 was most conservative at low replacement ratios, underestimating the capacity by 23% at *r* = 10%. Furthermore, for specimen src8, which contained no core concrete, the prediction errors of GB 50936-2014 and CECS 254:2012 were less than 2%, whereas CECS 159:2004 overestimated the capacity by 28.4%.

In summary, GB 50936-2014 and CECS 254:2012 demonstrate satisfactory overall prediction accuracy and stability for estimating the eccentric compression capacity of RuCFST columns. However, systematic deviations remain under conditions of high slenderness, low rubber replacement ratio, or the absence of initial imperfections. CECS 159:2004 tends to yield unsafe predictions overall, while DBJ13-51-2010 is overly conservative. The findings highlight that existing codes do not adequately consider the coupled influence of the rubber aggregate replacement ratio on the confinement effect and member stability. Future work should focus on developing specialized calculation methods for RuCFST members to improve prediction accuracy and ensure appropriate safety margins.

#### 3.5.2. International Design Provisions

**Figure 13:**
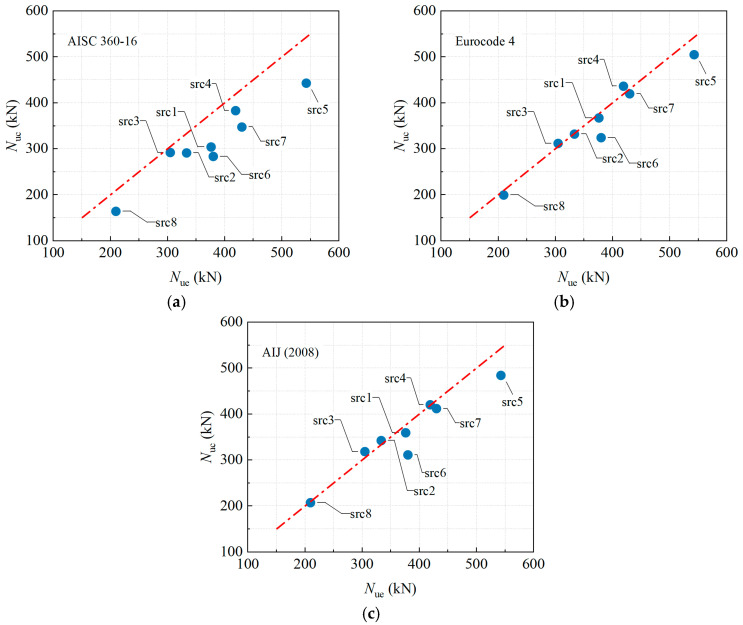
Comparison between calculated strength by codes of different countries and test results. (**a**) AISC 360-16 [34]. (**b**) Eurocode 4 [35]. (**c**) AIJ (2008) [36].

Table 5 compares the *N*_ue_ with the *N*_uc_ calculated according to AISC 360-16 [34], Eurocode 4 [35], and AIJ (2008) [36]. Figure 13 provides a direct visual comparison of the predicted versus experimental *N*_ue_ for each specimen, facilitating an intuitive assessment of the predictive performance of the three codes.

The AISC 360-16 code yields the most conservative predictions, with a mean *N*_uc_/*N*_ue_ ratio of 0.838 and a COV of 0.085. Its conservatism is particularly pronounced for specimens with high eccentricity, specifically src1, src6, and src7 with *e*_0_ = 40 mm, showing ratios between 0.746 and 0.808, and for the empty steel tube specimen src8 with a ratio of 0.783. This indicates that the AISC code provides the largest safety margin when accounting for adverse factors such as eccentric loading, initial geometric imperfections, and the absence of concrete infill.

Eurocode 4 has the best prediction accuracy and the least amount of scatter, with a mean *N*_uc_/*N*_ue_ ratio of 0.968 and a COV of only 0.061. The predicted ratios for all other specimens fall within the narrow range of 0.952 to 1.041, except for slight underestimations observed in the axial compression specimen with natural aggregate (src5), which has a ratio of 0.930, and the specimen with 10% rubber content (src6), which has a ratio of 0.853. This indicates excellent reliability and consistency across various slenderness ratios, eccentricities, and rubber content levels.

The overall performance of the AIJ (2008) code lies between the other two, with a mean *N*_uc_/*N*_ue_ ratio of 0.961 and a COV of 0.077. A slight overestimation is noted for the more slender specimens, specifically src2 and src3 with *λ* = 60 and 80, respectively, showing ratios of 1.027 and 1.044, while the code maintains satisfactory accuracy under conditions combining high eccentricity and initial imperfections.

In summary, Eurocode 4 provides the most accurate predictions with the least scatter for the load-bearing capacity of eccentrically loaded RuCFST members, making it well-suited for the design of such components. While AISC offers the greatest safety margin, its excessive conservatism may lead to material inefficiency. The accuracy of AIJ falls between the other two codes, though it carries a slight risk of overestimation for slender members. This comparative analysis indicates that, among the current international codes considered, Eurocode 4 is the most appropriate for calculating the load-bearing capacity of RuCFST columns.

## 4. Conclusions

This study conducted eccentric compression tests on eight groups of square specimens (two identical replicates per group), including six RuCFST groups with fine aggregate replacement ratios of 10% and 20%, one conventional CFST group, and one hollow steel tube group. The main conclusions are as follows:

(1) Increasing *λ* diminishes *N*_ue_ and *SI*. The *DI* reaches its peak at *λ* = 60, where the energy dissipation effect of rubber is most pronounced.

(2) Reducing *e*_o_ significantly enhances both load-bearing capacity and stiffness. The inclusion of rubber does not alter the fundamental mechanism whereby eccentricity governs performance degradation.

(3) Incorporating rubber particles reduces mechanical properties. At *r* = 10%, *N*_ue_ decreases by approximately 12.5%; a further increase to 20% yields no significant additional reduction, as the more uniform particle distribution optimizes stress transfer, partially counteracting the strength loss.

(4) Confining the rubberized concrete within the steel tube markedly enhances load-bearing capacity and stiffness, with a marginal increase in ductility. The primary enhancement mechanism is the core concrete delaying local buckling of the steel tube.

(5) Design codes GB 50936-2014, CECS 254:2012, and Eurocode 4 yield accurate predictions, with Eurocode 4 exhibiting the lowest scatter, whereas AISC 360-16 is conservative. It should be noted that none of the codes discussed in this study account for the influence of rubber on the structural behavior of the concrete core.

The findings are limited to square cross-sections, moderate rubber replacement ratios, and a single steel grade; future work should explore circular sections, higher replacement levels, cyclic loading for seismic performance, and bond–slip behavior through push-out tests.

In summary, this work provides crucial experimental evidence and clear design recommendations that strongly support the safe application of waste tire rubber in load-bearing composite columns, contributing to more sustainable and resilient infrastructure.

## Figures and Tables

**Table 1:** Parameters of test specimens.

Specimens ID	*B* × *t* × *L* (mm)	*λ*	*e*_0_ (mm)	*e*_0_/*B*	*r* (%)
src1	140 × 2.72 × 1400	40	40	0.286	20
src2	140 × 2.72 × 2100	60	40	0.286	20
src3	140 × 2.72 × 2800	80	40	0.286	20
src4	140 × 2.72 × 1400	40	20	0.143	20
src5	140 × 2.72 × 1400	40	0	-	20
src6	140 × 2.72 × 1400	40	40	0.286	10
src7	140 × 2.72 × 1400	40	40	0.286	0
src8	140 × 2.72 × 1400	-	40	0.286	-

*Note:* The nominal slenderness ratio *λ* was assigned only to the concrete-filled specimens (src1–src7), while the hollow steel tube specimen src8 was not assigned a *λ* value, as its stability is primarily governed by local buckling rather than overall buckling.

**Table 2:** Measured mechanical properties of the steel.

*f*_y_ (MPa)	*f*_u_ (MPa)	*E*_s_ (GPa)	*μ*_s_
260	350	176	0.3

**Table 3:** Mix proportions and mechanical properties of concrete with varying rubber content.

*r*	Water (kg/m^3^)	Fly Ash (kg/m^3^)	Cement (kg/m^3^)	Aggregate (kg/m^3^)	Sand (kg/m^3^)	Rubber (kg/m^3^)	*f*_cu_ (MPa)	*f*_c_^′^ (MPa)	*E*_c_ (GPa)
0%	164	72	340	1138	663	0	43.40	25.76	27.7
10%	164	72	340	1138	597	30	38.20	22.41	26.0
20%	164	72	340	1138	530	60	34.60	20.10	24.7

*Note*: Cube specimens: 100 mm × 100 mm × 100 mm; prism specimens: 100 mm × 100 mm × 300 mm. All values are averages of three specimens tested in accordance with GB/T 50081-2019.

**Table 4:** Comparison of calculated strength by different codes of China and test peak loads.

Specimen ID	*N*_ue_ (kN)	GB 50936-2014 [30]		CECS 254:2012 [31]		CECS 159:2004 [32]		DBJ 13-51-2010 [33]	
		*N*_uc_ (kN)	*N*_uc_/*N*_ue_	*N*_uc_ (kN)	*N*_uc_/*N*_ue_	*N*_uc_ (kN)	*N*_uc_/*N*_ue_	*N*_uc_ (kN)	*N*_uc_/*N*_ue_
src1	376.19	361.52	0.961	369.42	0.982	350.23	0.931	346.85	0.922
src2	333.33	344.00	1.032	356.33	1.069	307.66	0.923	318.00	0.954
src3	304.76	319.69	1.049	328.53	1.078	284.65	0.934	257.83	0.846
src4	419.05	422.40	1.008	428.69	1.023	401.03	0.957	396.00	0.945
src5	542.86	486.95	0.897	496.17	0.914	562.95	1.037	454.92	0.838
src6	380.00	313.12	0.824	314.64	0.828	432.44	1.138	292.60	0.770
src7	430.00	414.09	0.963	421.83	0.981	486.76	1.132	397.32	0.924
src8	209.52	208.26	0.994	212.87	1.016	269.02	1.284	200.30	0.956
Mean		-	0.966	-	0.986	-	1.042	-	0.894
Standard deviation		-	0.074	-	0.083	-	0.132	-	0.068
Variability coefficient		-	0.077	-	0.084	-	0.127	-	0.076

**Table 5:** Comparison of calculated strength by codes of different countries and test peak loads.

Specimen ID	*N*_ue_ (kN)	AISC 360-16 [34]		Eurocode 4 [35]		AIJ (2008) [36]	
		*N*_uc_ (kN)	*N*_uc_/*N*_ue_	*N*_uc_ (kN)	*N*_uc_/*N*_ue_	*N*_uc_ (kN)	*N*_uc_/*N*_ue_
src1	376.19	303.96	0.808	367.16	0.976	359.26	0.955
src2	333.33	291.00	0.873	332.00	0.996	342.33	1.027
src3	304.76	291.96	0.958	311.77	1.023	318.17	1.044
src4	419.05	383.01	0.914	436.23	1.041	420.31	1.003
src5	542.86	442.97	0.816	504.86	0.930	484.23	0.892
src6	380.00	283.48	0.746	324.14	0.853	311.22	0.819
src7	430.00	347.44	0.808	419.68	0.976	411.94	0.958
src8	209.52	164.05	0.783	199.46	0.952	207.22	0.989
Mean		-	0.838	-	0.968	-	0.961
Standard deviation		-	0.071	-	0.059	-	0.074
Variability coefficient		-	0.085	-	0.061	-	0.077

## Data Availability

The original contributions presented in this study are included in the article. Further inquiries can be directed to the corresponding author.
